# Tuberculosis in children and adolescents with rheumatic diseases using biologic agents: an integrative review

**DOI:** 10.1590/1984-0462/2024/42/2022084

**Published:** 2023-07-10

**Authors:** Lenita de Melo Lima, Rafaela Baroni Aurilio, Adriana Rodrigues Fonseca, Ana Alice Amaral Ibiapina Parente, Maria de Fátima Bazhuni Pombo Sant’Anna, Clemax Couto Sant’Anna

**Affiliations:** a Instituto de Puericultura e Pediatria Martagão Gesteira, Rio de Janeiro, RJ, Brazil.; b Universidade Federal do Rio de Janeiro, Rio de Janeiro, RJ, Brazil.

**Keywords:** Tuberculosis, Latent tuberculosis, Biologic agents, Rheumatic diseases, Children, Adolescents, Tuberculose, Tuberculose latente, Fatores biológicos, Doenças reumáticas, Crianças, Adolescentes

## Abstract

**Objective:**

To conduct a bibliographic review on tuberculosis (TB) disease in children and adolescents with rheumatic diseases, being managed with biologic therapy.

**Data source:**

An integrative review with a search in the U.S. National Library of Medicine and the National Institutes of Health (PubMed) using the following descriptors and Boolean operators: ([“*tuberculosis*”] AND ([“children”] OR [“adolescent”]) AND [“rheumatic diseases”] AND ([“tumor necrosis factor-alpha”] OR [“etanercept”] OR [“adalimumab”] OR [“infliximab”] OR [“biological drugs”] OR [“rituximab”] OR [“belimumab”] OR [“tocilizumab”] OR [“canakinumab”] OR [“golimumab”] OR [“secukinumab”] OR [“ustekinumab”] OR [“tofacitinib”] OR [*“*baricitinib”] OR [“anakinra”] OR [“rilonacept”] OR [“abatacept”]), between January 2010 and October 2021.

**Data synthesis:**

Thirty-seven articles were included, with the total number of 36,198 patients. There were 81 cases of latent tuberculosis infection (LTBI), 80 cases of pulmonary tuberculosis (PTB), and four of extrapulmonary tuberculosis (EPTB). The main rheumatic disease was juvenile idiopathic arthritis. Among LTBI cases, most were diagnosed at screening and none progressed to TB disease during follow-up. Of the TB cases using biologics, most used tumor necrosis factor-alpha inhibitors (anti-TNFα) drugs. There was only one death.

**Conclusions:**

The study revealed a low rate of active TB in pediatric patients using biologic therapy. Screening for LTBI before initiating biologics should be done in all patients, and treatment, in cases of positive screening, plays a critical role in preventing progression to TB disease.

## RESUMO

**Objetivo:**

Fazer um levantamento bibliográfico referente à tuberculose (TB) em crianças e adolescentes com doenças reumáticas, em uso de imunobiológicos.

**Fonte de dados:**

Revisão integrativa com busca na base United States National Library of Medicine (PubMed) utilizando os descritores e operadores booleanos: ([“*tuberculosis*”] AND ([“children”] OR [“adolescent”]) AND [“rheumatic diseases”] AND ([“tumor necrosis fator-alpha”] OR [“etanercept”] OR [“adalimumab”] OR [“infliximab”] OR [“biological drugs”] OR [“rituximab”] OR [“belimumab”] OR [“tocilizumab”] OR [“canakinumab”] OR [“golimumab”] OR [“secukinumab”] OR [“ustekinumab”] OR [“tofacitinib”] OR [*“*baricitinib”] OR [“anakinra”] OR [“rilonacept”] OR [“abatacept”]), entre janeiro de 2010 e outubro de 2021.

**Síntese de dados:**

Trinta e sete artigos foram incluídos, com o total de 36.198 pacientes. Houve 81 casos de tuberculose latente (ILTB), 80 casos de tuberculose pulmonar (TBP) e quatro casos de tuberculose extrapulmonar (TBEP). A principal doença reumática foi a artrite idiopática juvenil. Entre os casos de ILTB, a maioria foi diagnosticada no rastreio e nenhum evoluiu para a TB. Dos casos de TB em uso de imunobiológicos, a maioria utilizava fármacos antiTNFα. Houve somente um caso de óbito.

**Conclusões:**

O estudo demonstrou baixa taxa de TB nos pacientes pediátricos em uso de imunobiológicos. O rastreio para ILTB antes do início da terapia com agentes biológicos deve ser realizado em todos os pacientes, e o tratamento, nos casos de rastreio positivo, é importante para evitar a progressão para TB doença.

## INTRODUCTION

Despite advances in the diagnosis and treatment of tuberculosis (TB), the disease remains among the ten leading causes of death in the world.^1^ It is estimated that 10 million people became ill due to TB in 2019 and that there were 1.2 million deaths in individuals with TB not infected with the human immunodeficiency virus (HIV) that same year.^1^

Children under the age of 15 accounted for 12% of reported TB cases in 2019,^1^ comprising more than a fifth of TB patients in high-incidence countries and accounting for 8–20% of TB-related deaths in those countries.^1^

Rheumatic diseases are a heterogeneous group of diseases characterized by alterations of the musculoskeletal system and systemic manifestations, including autoimmune connective tissue diseases, auto-inflammatory diseases, and vasculitis. Patients with rheumatic diseases exhibit a higher frequency of TB than the general population (2–10 times in adults), which may be associated with immunosuppression linked to the underlying disease, as well as due to the use of immunosuppressive medications, such as corticosteroids and biologics.^2^

The objective of this study was to conduct a bibliographic review, referring to TB disease in children and adolescents with rheumatic diseases, being managed on biologic agents.

## LITERATURE REVIEW

For the bibliographic review, referring to TB in children and adolescents with rheumatic diseases, a search was performed in the U.S. National Library of Medicine and the National Institutes of Health (PubMed) using the following descriptors and Boolean operators: ([“*tuberculosis*”] AND ([“*children*”] OR [“*adolescent*”]) AND [“rheumatic diseases”] AND ([“*tumor necrosis factor-alpha*”] OR [“*etanercept*”] OR [“*adalimumab*”] OR [“*infliximab*”] OR [“*biological drugs*”] OR [“*rituximab*”] OR [“*belimumab*”] OR [“*tocilizumab*”] OR [“*canakinumab*”] OR [“*golimumab*”] OR [“*secukinumab*”] OR [“*ustekinumab*”] OR [“*tofacitinib*”] OR [*“baricitinib*”] OR [“*anakinra*”] OR [“*rilonacept*”] OR [“*abatacept*”]). The PubMed database was adopted because it is the main source of consultation in the medical field.

The inclusion criteria defined for the selection of articles were studies published in English, Portuguese, and Spanish on pulmonary TB (PTB) and extrapulmonary TB (EPTB) in children and adolescents aged 0–19 years with rheumatologic diseases using biologic drugs. The time period of the bibliographic search was from January 2010 to October 2021. Exclusion criteria were studies that 1) addressed only latent tuberculosis infection (LTBI) and 2) included a narrative literature review.

Initially, the titles and abstracts were evaluated. Following the initial screening, the selected articles were read in full, and the studies to be included in the review were finally chosen. After the complete reading of the articles, a direct search of the bibliographic references was performed by author and title in the PubMed database in order to find articles omitted from the initial search and relevant to the subject. To minimize biases, the search, evaluation, and selection of studies were carried out by two independent reviewers. When there was disagreement, a third reviewer was consulted and, after a consensus discussion, with the agreement of all, the articles to be included on the review were selected.

In the present review, some items of the articles were evaluated to assess their quality: objective; adequate methodology for the proposed objective; detailed data collection; rigor in data analysis; presentation and discussion of results; and research contributions and limitations. Articles that fulfilled all the criteria were able to be included in the review.

## RESULTS

**Figure 1.**
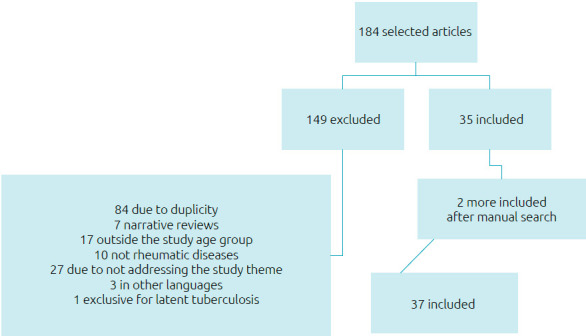
Flowchart: Bibliographic search on tuberculosis in children and adolescents with rheumatic diseases.

Thirty-seven studies were included.^3–39^ From the bibliographic references of the works read in full, two articles were included through a direct search by author and title in the PubMed database. Figure 1 illustrates the bibliographic search.

Among the 37 references, most studies were prospective (15/37) and retrospective (10/37), followed by case reports (4/37).

The total number of patients investigated was 36,198, with 30,246 pediatric rheumatologic patients using biologic drugs. There were 81 cases of LTBI, 80 cases of PTB, and four cases of EPTB. In 34 studies, the disease evaluated was juvenile idiopathic arthritis (JIA), and in three, in addition to JIA, systemic lupus erythematosus (SLE), juvenile dermatomyositis (JDM), familial Mediterranean fever, spondyloarthritis, vasculitis, collagen diseases and auto-inflammatory syndromes.

Table 1 ^3–39^ shows the authors, year of publication, type of studies — with the number of pediatric rheumatologic patients using biologic drugs — and population — age and rheumatic diseases.

**Table 1.** Articles included in the bibliographic search for tuberculosis in children and adolescents with rheumatic diseases being treated with biologic drugs.

Author/year	Type of study (n*)	Rheumatic disease/age
Ruperto et al.^3^ 2010	Randomized double-blind (58)	JIA; 6–17 y.
Kilic et al.^4^ 2012	Retrospective cohort (144)	JIA^†^; 12.25 (4.08–19.41) y.
Sevcic et al.^5^ 2011	Prospective observational (72)	JIA; 10.0 (±3.4) y.
Zuber et al.^6^ 2011	Prospective observational (188)	JIA; 3.5–18 y.
Imagawa et al.^7^ 2012	Prospective multicenter (19)	JIA; mean age of 11.6 y.
Imagawa et al.^8^ 2012	Prospective multicenter (25)	JIA; 4–17 y.
Lovell et al.^9^ 2013	Prospective (23)	JIA; mean age of 14 y.
Atikan et al.^10^ 2016	Prospective (71)	JIA; 3.5–18 y.
Al-Mayouf et al.^11^ 2016	Retrospective (134)	JIA^‡^; mean age of 9.3 y.
Hsin et al.^12^ 2015	Case-control (111)	JIA; 10.5 (±3,6) y.
Tarkiainen et al.^13^ 2015	Retrospective, multicenter and observational (348)	JIA; 10.8 (2.17–19.16) y.
Walters et al.^14^ 2015	Prospective cohort (20)	JIA; 1–21 y.
Guerrero-Laleona et al.^15^ 2017	Case report (1)	JIA; a 9-year-old patient.
Becker and Horneff^16^ 2017	Prospective and observational (1897)	JIA; 7.4–14.9 y.
Klotsche et al.^17^ 2016	Prospective observational (1734)	JIA; mean age of 12.6 y.
Verazza et al.^18^ 2016	Retrospective and cross-sectional (1038)	JIA; median age of 10.1 y.
Saini et al.^19^ 2016	Retrospective (10)	JIA; median age of 7.3 y.
Constantin et al.^20^ 2016	Prospective (127)	JIA; mean age of 8.6 y.
Brunelli et al.^21^ 2018	Longitudinal (107)	JIA; 14.6 (± 5.7) y.
Bal et al.^22^ 2017	Case report (1)	JIA; a 13-year-old adolescent.
Swart et al.^23^ 2018	Two-way observational (12,264)	JIA; 2.4-11.7 y.
Ozere et al.^24^ 2018	Case report (1)	JIA; a 9-year-old patient.
Aeschlimann et al.^25^ 2019	Systematic literature review (1607)	JIA; 8.7–15.3 y.
Cabrera et al.^26^ 2019	Observational, retrospective, multicenter (813)	JIA^§^; 9.4 (± 3,6) y.
Choi et al.^27^ 2018	Retrospective (83)	JIA; mean age of 12.6 y.
Aygun et al.^28^ 2019	Prospective (307)	JIA; 9 (±4.33)y.
Dumaine et al.^29^ 2020	Retrospective (677)	JIA; mean age of 7.8 y.
Nagy et al.^30^ 2019	Meta-analysis (2,130)	JIA; 2-18 y.
Foeldvari et al.^31^ 2019	Prospective (109)	JIA; 13.3 (±4.5) y.
Diener and Horneff^32^ 2019	Meta-analysis (2176)	JIA; 2-19 y.
Vaidya et al.^33^ 2019	Case report (1)	JIA; 14-year-old adolescent.
Yazilitaş et al.^34^ 2019	Retrospective – case series(11)	JIA; 3–8 y.
Cakan et al.^35^ 2019	Retrospective cohort (123)	JIA; mean age not related.
Balci et al.^36^ 2020	Retrospective (162)	JIA; 10.5 (±4.3) y.
Brunner et al.^37^ 2020	Prospective, observational and multicenter (537)	JIA; 2–17 y.
Brunner et al.^38^ 2020	Prospective (392)	JIA; 2–17 y.
Armaroli et al.^39^ 2020	Prospective (2725)	JIA; +12.1 (±4.4)y.

JIA: juvenile idiopathic arthritis; y: years. *pediatric rheumatologic patients used biologic drugs; ^†^and familial Mediterranean fever and chronic idiopathic uveitis; ^‡^and spondyloarthritis, systemic lupus erythematosus, auto-inflammatory syndromes, juvenile dermatomyositis, vasculitis and others; ^§^and auto-inflammatory diseases, uveitis, inflammatory bowel disease-related arthritis, vasculitis, connective tissue diseases, chronic multifocal osteomyelitis, Behçet’s disease, unclassified auto-inflammatory diseases, Blau syndrome, SAPHO syndrome, Castleman disease, and immune dysregulation.

Among the 81 cases of LTBI described, most (73/81) were diagnosed at screening, before therapy with a biologic agent was initiated, and no case progressed to TB disease during follow-up. Only 7/81 of the cases were diagnosed during treatment with biologics: 5/7 treated with etanercept (ETA), 1/7 with infliximab (IFX), and 1/7 with sequential use of IFX, adalimumab (ADA), and ETA. All had been screened before the start of the biologic agent. One case of LTBI occurred in a patient using methotrexate (MTX), with previous therapy with ETA. In this case, there is no report of pre-biologic screening.

Table 2 shows TB disease and LTBI cases according to biologic agents and screening before biologic therapy.

**Table 2.** Cases of tuberculosis disease and latent tuberculosis infection according to biologic agents and screening before biologic therapy.

Reference	Biologic agents	Cases of TB active or LTBI*	Screening^†^
12	ETA, ADA, IFX, ANA, CAN, TCZ	2 PTB	Yes
16	ETA, ADA, IFX, TCZ, ANA, CAN	0	Yes
18	ETA, IFX, ETA+IFX, IFX+ADA, ETA+ADA	7 LTBI	Yes
20	Anti-TNFα	1 PTB	No
21	ETA, ADA	1 EPTB	Yes
22	ETA, ADA, CAN, TCZ	0	Yes
23	ETA, IFX, ADA, GOL, RTX, ANA, TCZ, ABA	0	No
24	ETA, ADA	0	No
25	ETA, ADA, IFX, ABA, TCZ	1 PTB	Yes
26	ETA, ADA, ABA	1 EPTB	No
27	ETA, ADA, IFX, GOL, TCZ, ABA, ANA, CAN, RTX, CER	17 PTB	No
28	ADA	1 EPTB	Yes
29	ETA, ADA, GOL, IFX, CAN, ANA, TCZ, ABA, RTX	0	No
30	ADA, ETA, GOL, IFX	0	Yes
31	ETA, ADA, ETA->ADA, ADA->CER	0	Yes
32	ETA, ETA->ADA	0	Yes
33	ABA	0	Yes
34	ETA, ADA, IFX, GOL, TCZ, ANA, CAN, RIL, ABA	0	No
35	ETA	1 PTB	No
36	ETA, ADA	0	No
37	ETA	1 PTB	No
38	ETA, TCZ, ABA	0	Yes
39	ETA	0	No
40	ETA	0	No
41	ETA, ADA, IFX, TCZ, CAN, ANA	2 PTB	Yes
42	ADA, ETA, GOL, IFX, ABA, TCZ	1 PTB	No
43	ETA, ADA, IFX, GOL	0	No
44	ETA, IFX, ADA, ANA, ABA	0	Yes
45	ADA	0	No
46	ADA	0	Yes
47	ADA	1 EPTB	Yes
48	TCZ	0	Yes
49	TCZ	0	No
50	RIL	0	Yes
51	ABA	0	No
52	ETA, ADA, IFX, ANA, RTX, TCZ, ABA	0	Yes
53	ETA	0	No

TB: tuberculosis; LTBI: latent tuberculosis infection; ETA: etanercept; ADA: adalimumab; IFX: infliximab; ANA: anakinra; CAN: canakinumab; TCZ: tocilizumab; PTB: pulmonary tuberculosis; TNFα: tumor necrosis factor-alpha; LTBI: latent tuberculosis infection; EPTB: extrapulmonary tuberculosis; GOL: golimumab; RTX: rituximab; ABA: abatacept; CER: certolizumab; RIL: rilonacep. *during biologic therapy; ^†^before biologic therapy.

Among the cases of PTB, 2/80 were diagnosed during the initial screening and 52/80 were in pediatric patients not using biologic drugs (34 in a control group without immunosuppressants and 18 who used non-biological immunosuppressive drugs). The other 26/80 cases of PTB were in patients using biologic agents. Among the 26, 21 were being treated with tumor necrosis factor-alpha inhibitors (anti-TNFα), two were using canakinumab, and three were using unspecified medications outside the anti-TNFα class.

**Figure 2.**
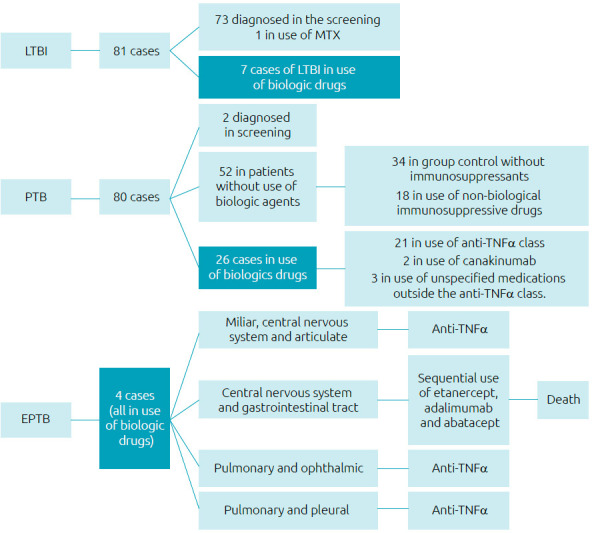
Flowchart: Summary of latent infection, pulmonary and extrapulmonary tuberculosis cases found in the review.

LTBI: latent tuberculosis infection; MTX: methotrexate; PTB: pulmonary tuberculosis; EPTB: extrapulmonary tuberculosis; TNFα: tumor necrosis factor-alpha;.

Of the four EPTB cases, 1/4 was miliary, central nervous system, and articular; 1/4, central nervous system and gastrointestinal tract; 1/4, pulmonary and ophthalmic; and 1/4, pulmonary and pleural. Three of the patients were being managed with anti-TNFα and one was sequentially using ETA, ADA, and abatacept, with a diagnosis of EPTB while using abatacept. The latter had a fatal outcome after complications of central nervous system involvement and was the only death reported in all the included studies. Figure 2 summarizes the cases of LTBI, PTB, and EPTB identified in the review.

Studies that evaluated the safety of biologic drugs and reported their findings on TB were included. Seventeen articles (total of 8,492 patients) did not report any cases of active TB or LTBI and also pointed to JIA as the most frequent disease.^3,5,7–9,13,14,16,17,19,20,26,27,29,31,37,38^ Most (10/17) were studies with anti-TNFα medications.^3,5,8,14,16,17,20,26,31,37^ There were only eight specific papers on TB in children with rheumatic diseases using biologics. One was a case-control study^12^ that evaluated the risk of TB in children with JIA in Taiwan, two were retrospective studies^4,35^ that evaluated the safety of anti-TNFα/biologics in relation to TB, one was a prospective study^10^ that evaluated TB during treatment with biologic agents in a population vaccinated with bacillus Calmette-Guérin (BCG), and four were case reports.^15,22,24,33^

Screening for TB before starting biologic therapy was performed in just over half (19/37) of the studies.^3–5,8–11,14,15,19,21,24,27,28,30,33–36^ Of the 30 cases of TB disease (PTB: 26, EPTB: 4), 8/30 occurred in patients who had been screened before biologic therapy and 22/30 in those not screened. Of the eight specific studies on TB, screening was conducted in six; of the two studies in which there was no report on screening for TB, one is the case that progressed to death.

## DISCUSSION

In Brazil, the diagnosis of PTB in children and adolescents is based on a scoring system of the Ministry of Health — Brazil.^40^ This system evaluates clinical, radiological, and epidemiological data and does not involve bacteriological confirmation, which is known to be challenging in children.^40^ The difficulty of bacteriological isolation in children occurs because the disease is paucibacillary in this age group and also because of the difficulty in collecting respiratory specimens.^40^

On chest radiography, signs suggestive of active TB are considered: mediastinal and/or lymph node enlargement, alveolar opacifications, pleural effusion, miliary nodules, and cavitations.^41^

The World Health Organization defines latent *Mycobacterium tuberculosis* infection or latent TB infection (LTBI) as a state of persistent immune response to stimulation by *Mycobacterium tuberculosis* (*M. tb*) antigens without evidence of clinical manifestations of active TB.^1,42^

In cases of LTBI, despite the absence of symptoms, there is a risk that patients with rheumatic diseases will develop TB disease, especially in the first two years after the primary infection.^1,42,43^ If LTBI is not diagnosed and treated appropriately, activation occurs in 5–10% of cases.^1,42,43^

There are two tests available for the diagnosis of LTBI, the tuberculin skin test (TST), and the interferon-gamma release assay tests (IGRAs). TST is performed by intradermal injection, using the Mantoux method, whereby purified protein derivative (PPD) detects previous infection with *M. tb* or other mycobacteria, and vaccination with BCG. TST may, therefore, produce a false-positive result because it is not specific to *M. tb.* ^44^ IGRAs are more specific tests because they detect the immune response against *M. tb* antigens that are not present in BCG or other mycobacteria.^44^ However, IGRAs are costlier than the TST and must be performed in a laboratory.^45^

A limitation associated with TST and IGRAs is related to the immunological status of patients. TST and IGRAs can be affected by severe immunosuppression in patients before starting treatment.^10^ Elevated levels of tumor necrosis factor-alpha (TNFα) in rheumatologic diseases reduces the cytokine response, with recovery after initiation of anti-TNFα therapy. One study^46^ reported conversion of TST to positive one year after the first screening, in 30% of patients who started treatment with anti-TNFα, which could be explained by the restoration of suppressed immune reactivity against TB antigens, with a decrease in the underlying disease activity.^10^ Children below five years of age may also have undetermined IGRAs.^47^

From 1975 onwards, there has been the development of biologic drugs, which are medications that act on cell signaling or interaction processes, resulting in the activation and/or regulation of the immune response.^48^ Biologic drugs are derived from living organisms produced using molecular biology techniques, which act as monoclonal antibodies or receptor or cytokine antagonists, hence the term “biologic” or “immunobiologic”.^48^

Table 3 summarizes the main biologic agents approved or being investigated for the treatment of pediatric rheumatic diseases and their mechanism of action.^49^

**Table 3.** Biologic drugs used for pediatric rheumatic diseases.^11^

Drug name	Target	Drug class
Etanercept	TNFα	Dimeric fusion protein
Infliximab		Chimeric monoclonal antibody
Adalimumab		Fully human monoclonal antibody
Golimumab*		Fully human monoclonal antibody
Rituximab	B-Cell CD20	Chimeric monoclonal antibody
Belimumab	BLyS	Fully human monoclonal antibody
Tocilizumab	IL6	Humanised monoclonal antibody
Canakinumab	IL1	Fully human monoclonal antibody
Anakinra^†^		Fully human IL-1 receptor antagonist
Rilonacept^†^		Fusion protein
Abatacept	CTLA4	Fully human fusion protein
Secukinumab*	IL17	Fully human monoclonal antibody
Ustekinumab*	IL12/23	Fully human monoclonal antibody
Tofacitinib*	JAK	Small molecular inhibitor
Baricitinib*		Small molecular inhibitor

BLyS: B-lymphocyte stimulator; CTLA-4: cytotoxic T-lymphocyte-associated antigen 4; IL: interleukin; JAK: Janus kinase; TNF: tumor necrosis factor. *Under investigation in pediatrics rheumatic diseases; ^†^Unavailable in Brazil.

In chronic inflammatory diseases, high concentrations of TNFα are produced, leading to excessive inflammation and damage to the body.^28,50^ For example, children with JIA or inflammatory bowel disease exhibit high levels of pro-inflammatory cytokines in peripheral blood, synovial fluid, or gastrointestinal mucosa.^28,50^ Anti-TNFα drugs prevent excessive inflammation and the consequent tissue damage.^50^

Among the biologic drugs, those that act on TNFα were developed first and tested in adults with rheumatoid arthritis, with positive results. This encouraged their application in other diseases, eventually expanding their use to pediatrics, particularly in cases that are refractory to traditional medications.^51^

ETA was the first anti-TNFα drug approved for the treatment of JIA in 1998.^52^ ADA and tocilizumab (TCZ) were the second and third biologics, respectively, used in patients with JIA.^35^

TNFα is a pro-inflammatory cytokine secreted by monocytes, macrophages, and T lymphocytes and develops an important role in the immune response against *M. tb,* ^2,52,53^ particularly in the formation and maintenance of granuloma integrity.^52,53^ TNFα works synergistically with interferon-gamma, increases the expression of intercellular adhesion molecules (essential for granuloma maintenance), and stimulates the production of the bactericidal compound from intermediates of nitrogen and oxygen.^4,50^ Therefore, the granuloma, composed of differentiated macrophages and lymphocytes, restricts the growth and spread of *M. tb*, resulting in a dynamic balance between pathogen and host and the induction of LTBI. Blocking this cytokine impedes the immune system’s ability to contain *M. tb* within granulomas.^50^

Exposure to these drugs has been associated with LTBI reactivation, even after discontinuation of treatment, with progression to TB disease.^2,52,53^ In some cases, extrapulmonary manifestations or disseminated forms of TB develop.^52^

Since the emergence of biologic agents, there has been an increase in the risk of infections, and patients should be kept under infection surveillance.^10,16,22,35^ Before starting biologic drugs, serious infections such as hepatitis B and C, HIV, and TB (active and LTBI) should be excluded or adequate therapeutic planning and treatment of these conditions undertaken whenever possible, before initiating biological therapy.^54^

Cakan et al. showed that screening with TST and chest X-ray, associated with treatment for LTBI with isoniazid for six months, in cases of TST ≥5 mm, would be sufficient to protect against active TB before initiating the biologic agent.^35^

Studies suggest that IFX and ADA increase the risk of TB compared to ETA.^4,12,15^ This difference may occur due to the differences in affinity of the receptors of the drugs; IFX has affinity for the TNF 1 receptor and ETA for the TNF 2 receptor.^4^ The TNF 2 receptor develops a less significant role in defending against TB.^4^ IFX and ADA reduce gamma interferon production by 65–70%, in contrast to ETA, which causes almost no change in gamma interferon.^4^

In turn, interleukin-1 (IL1) appears to be involved in late hypersensitivity to *M. tb*. However, this does not play a fundamental role in infection control, which may explain why there is minimal or no risk of developing TB in patients treated with anti-IL1 drugs.^10^

Each country determines its guidelines for LTBI screening; some guide the use of TST and IGRAs (European Society of Rheumatology 2010 and Australian Rheumatology Society 2011).^44^ The American guideline recommends screening for all patients scheduled to initiate therapy with biologics, such as IGRAs or TST.^44^ The American College of Rheumatology recommends that patients with JIA be screened for LTBI before initiating biologics.^10^

The evaluation of LTBI in Brazil includes the performance of TST, chest X-ray, and epidemiological history of close contact with TB, which should be performed before starting biological therapy. In patients with no clinical-radiological signs of active TB and TST ≥5 mm or history of contact with TB, LTBI treatment should be performed, and a biologic drug should be initiated one month after the start of the LTBI treatment, in adults.^43^

Screening for LTBI and administering treatment before starting biologics have proven to be effective in preventing the TB activation.^14,22,35^ Physicians and patients should be aware of the risks related to TB in order to initiate investigation for the disease as early as possible in case of suggestive signs/symptoms or an epidemiological history of contact with TB. In the study by Atikan et al., screening for TB is strongly recommended before starting therapy with biologics, as well as annually, with chest radiography, TST, and/or IGRAs, in addition to clinical history, for all patients who continue to be managed on biologic therapy.^10^

However, the periodicity of screening for TB in the pediatric population using biologic therapy requires evaluation in further prospective studies and in countries with high rates of TB. However, in countries with a low rate of the disease, systematic repeat screening for TB in children using anti-TNFα drugs, after an initial negative test, may not be necessary.^23^

Among the included studies, the only case of death occurred in a patient sequentially using ETA (for seven years), ADA, and ABA (for three months each). The diagnosis of EPTB was made when using ABA, a fully humanized fusion protein, the activity of which occurs on antigen 4 associated with cytotoxic T lymphocytes. This drug is safe and is associated with a low rate of adverse effects. In this case, the fact that the patient used anti-TNFα therapy for a considerable time before starting ABA may be related to the severity of the TB infection because in patients using anti-TNFα drugs, TB cases can be more severe.

The present review of literature demonstrates the low rate of TB in pediatric patients using biologic therapy, which can be related to the screening and appropriate treatment of LTBI cases before initiating use of a biologic drug.

Biologics are drugs with tremendous value in the control of rheumatologic diseases in pediatric patients, either alone or in combination with other immunosuppressive medications, resulting in improved quality of life and prognosis.^55^

The articles included in the discussion demonstrate the relevance of the theme. Most of them were prospective, but had a small sample, which can be considered as a limitation. The source of the articles was PubMed, the main source of consultation in the medical field; however, other databases were not searched, which is another limitation of our study. In addition, this was an integrative review, not including statistical methods. A systematic literature review is suggested to facilitate a statistical and broader assessment of TB cases in pediatric patients treated with biologic agents.

As a conclusion, the study revealed a low rate of active TB in pediatric rheumatic patients using biologic therapy, which may be associated with screening and effective therapy for LTBI before the initiation of biologic agents. Screening for LTBI before initiating biologic therapy should be conducted in all patients, particularly in endemic areas for TB, such as Brazil. The treatment administered in cases of positive screening plays a critical role in preventing progression to TB disease.
